# Deletion of Fibroblast Growth Factor Receptor 2 from the Peri-Wolffian Duct Stroma Leads to Ureteric Induction Abnormalities and Vesicoureteral Reflux

**DOI:** 10.1371/journal.pone.0056062

**Published:** 2013-02-07

**Authors:** Kenneth A. Walker, Sunder Sims-Lucas, Valeria E. Di Giovanni, Caitlin Schaefer, Whitney M. Sunseri, Tatiana Novitskaya, Mark P. de Caestecker, Feng Chen, Carlton M. Bates

**Affiliations:** 1 Rangos Research Center, Children's Hospital of Pittsburgh of UPMC, Pittsburgh, Pennsylvania, United States of America; 2 Department of Medicine, Division of Nephrology, Vanderbilt University Medical Center, Nashville, Tennessee, United States of America; 3 Renal Division, Department of Internal Medicine, Washington University School of Medicine, St. Louis, Missouri, United States of America; 4 Division of Nephrology, Department of Pediatrics, University of Pittsburgh School of Medicine, Pittsburgh, Pennsylvania, United States of America; The University of Tennessee Health Science Center, United States of America

## Abstract

**Purpose:**

Pax3cre-mediated deletion of fibroblast growth factor receptor 2 (*Fgfr2*) broadly in renal and urinary tract mesenchyme led to ureteric bud (UB) induction defects and vesicoureteral reflux (VUR), although the mechanisms were unclear. Here, we investigated whether Fgfr2 acts specifically in peri-Wolffian duct stroma (ST) to regulate UB induction and development of VUR and the mechanisms of Fgfr2 activity.

**Methods:**

We conditionally deleted *Fgfr2* in ST (*Fgfr2^ST−/−^*) using *Tbx18cre* mice. To look for ureteric bud induction defects in young embryos, we assessed length and apoptosis of common nephric ducts (CNDs). We performed 3D reconstructions and histological analyses of urinary tracts of embryos and postnatal mice and cystograms in postnatal mice to test for VUR. We performed in situ hybridization and real-time PCR in young embryos to determine mechanisms underlying UB induction defects.

**Results:**

We confirmed that *Fgfr2* is expressed in ST and that *Fgfr2* was efficiently deleted in this tissue in *Fgfr2^ST−/−^* mice at embryonic day (E) 10.5. E11.5 *Fgfr2^ST−/−^* mice had randomized UB induction sites with approximately 1/3 arising too high and 1/3 too low from the Wolffian duct; however, apoptosis was unaltered in E12.5 mutant CNDs. While ureters were histologically normal, E15.5 *Fgfr2^ST−/−^* mice exhibit improper ureteral insertion sites into the bladder, consistent with the ureteric induction defects. While ureter and bladder histology appeared normal, postnatal day (P) 1 mutants had high rates of VUR versus controls (75% versus 3%, p = 0.001) and occasionally other defects including renal hypoplasia and duplex systems. P1 mutant mice also had improper ureteral bladder insertion sites and shortened intravesicular tunnel lengths that correlated with VUR. E10.5 *Fgfr2^ST−/−^* mice had decreases in *Bmp4* mRNA in stromal tissues, suggesting a mechanism underlying the ureteric induction and VUR phenotypes.

**Conclusion:**

Mutations in *FGFR2* could possibly cause VUR in humans.

## Introduction

Murine metanephric kidney development starts at E10.5 when an outgrowth from the Wolffian duct, the ureteric bud, is induced by the adjacent metanephric mesenchyme (MM) [1]. The UB induction site is constrained to its proper position by tailbud mesenchyme derived stroma (ST) lying between the Wolffian duct and MM [2]. Perturbed growth factor signaling within the ST such as haploinsufficiency of bone morphogenetic protein 4 (Bmp4) causes a shift in the UB induction site leading to urinary tract anomalies including ectopic ureters and renal hypoplasia [2]. In addition, aberrant ureteric induction sites are associated with abnormal positioning of the ureteral insertion site into the bladder and increased likelihood of vesicoureteral reflux in humans and animal models [3], [4].

VUR may be present in up to 2% of children and is associated with the development of reflux nephropathy, a leading cause of pediatric chronic kidney disease [5]. In addition to abnormal position of ureteral insertion into the bladder, VUR has been associated with a number of other defects including shortened intravesicular ureteral tunnel lengths [5], [6], [7] and bladder and ureteral muscle defects [8]. Despite some insights into pathophysiology, the genetics of VUR remain unclear. VUR follows a dominant inheritance pattern, however no single gene defect has been shown to cause the majority of VUR cases [5].

Fibroblast growth factor receptors 1 and 2 are critical for different stages and lineages of kidney development [9]–[17]. While conditional deletion of *Fgfr1* with the transgenic *Pax3cre* line in the peri-Wolffian duct stroma and MM resulted in no kidney defects, *Pax3cre*-mediated deletion of *Fgfr2* led to UB induction abnormalities and high rates of VUR [9], [10], [11]. Despite these data, it is unclear whether deletion of Fgfrs in the ST or the MM drives the abnormalities and what downstream targets mediate the actions of Fgfr2.

To determine whether loss of *Fgfr2* in the ST alters ureteric induction leading to VUR, we utilized a *Tbx18cre* mouse line [18] to delete *Fgfr2* expression in peri-Wolffian duct stroma but not in MM. E11.5 *Fgfr2^ST−/−^* mice exhibit aberrant ureteric induction with approximately 1/3 arising too high and 1/3 too low from the Wolffian duct. 3D imaging of E15.5 mutants revealed abnormal ureteral insertion sites into the bladder, consistent with the ureteric induction defects. P1 mutants had high rates of VUR associated with improper ureteral insertion sites into the bladder and shortened intravesicular tunnel lengths. Rarely *Fgfr2^ST−/−^* mice had other anomalies such as renal hypoplasia or duplex systems. Loss of *Fgfr2* in ST led to a decrease in *Bmp4* expression at E10.5 that is likely the reason for the ureteric induction defects.

## Materials and Methods

### Mice

*Tbx18cre^Tg^* ^/+^ mice that drive cre expression in the peri-Wolffian duct stroma [18] were bred with *Fgfr2^Lox/Lox^* mice, a gift from Dr. David Onritz [19], to produce *Tbx18^Tg^* ^/+^ *Fgfr2^Lox/Lox^* mice with deletion of *Fgfr2* in the ST (*Fgfr2^ST−/−^*). To detect cre expression, *Tbx18cre^Tg^* ^/+^ and *Fgfr2^ST−/−^* mice were bred with *Cag:RFP^Tg/+^* reporter mice (*Cag-Tbx18cre^Tg/+^* and *Cag-Fgfr2^ST−/−^*) to generate offspring in which red fluorescent protein (RFP) is expressed at the site of cre recombinase [20]. For embryonic studies, timed overnight matings were generated; when a vaginal plug was identified, noon of that day was deemed E0.5. All experiments were carried out with approval of the University of Pittsburgh Institutional Animal Care and Use Committee.

### Genotyping

DNA was extracted from tail clippings or embryonic tissues for PCR genotyping, as described [9]. The following genotyping primers were used to detect respective gene expression: *Tbx18cre* - 5′-CCATCCAACAGCACC TGGGCCAGCTCAACA-3′ and 5-CCACCATCGGTGCGGGAGATGTCCTTCACT-3′, *Fgfr2* - 5′-GTCAATTCTAAGCCACTGTCTGCC-3′ (wildtype allele), 5′-CTCCACTGATTACATCTAAAGAGC-3′ (floxed allele), CAG – 5′-AAG GGA GCT GCA GTG GAG TA-3′, 5′-CCG AAA ATC TGT GGG AAG TC-3′ (wildtype allele), 5′-GGC ATT AAA GCA GCG TAT CC-3′, 5′-CTG TTC CTG TAC GGC ATG G-3′ (Cag allele).

### Gene Expression Analysis

Whole-mount in situ hybridization (WISH) was performed on E10.5 control and *Fgfr2^ST−/−^* tissues as described [9]. Briefly, digoxigenin UTP-labeled antisense RNA probes were generated against *Bmp4* (Gene Accession number: NM007554.2), *Fgfr2* (NM201601.2) and *Ret* (NM001080780.1).

### Fluorescence Activated Cell Sorting (FACS)

Six E10.5 *Cag-Tbx18cre^Tg/+^* and *Cag-Fgfr2^ST−/−^* urogenital ridges were dissected and then manually disassociated with 0.3% collagenase into a cellular suspension in preparation for flow cytometry. *Tbx18cre^+ve^* cells were FACsorted from other cells via the presence of RFP. Following flow cytometry, cells from each genotype were pooled for subsequent Real-time PCR analysis.

### Real-time PCR

For quantitative analysis mRNA expression, six E10.5 *Fgfr2^ST−/−^* and control urogenital ridges, or pooled cells as mentioned previously, were collected and RNA extracted using a Mini-prep kit (Qiagen, Valencia, CA). To analyze gene expression, TaqMan® Gene Expression assays for *Fgfr2*, *Ret* and *Bmp4* and *Gapdh* (NM_008084.2; endogenous control) were utilized (Invitrogen, Carlsbad, CA). Quantitative real-time PCR was performed as described [21] on an Applied Biosystems ABI 7900 HT (Foster City, CA).

### Common nephric duct length, proliferation and apoptosis

To assess common nephric duct (CND) length E11.5 urogenital ridges from *Fgfr2^ST−/−^* and cre negative littermate control embryos were dissected into ice cold methanol, washed in PBS, blocked and stained whole mount with a pan-cytokeratin antibody (1∶10; Sigma Aldrich) followed by goat anti-mouse Alex Fluor 594 (1∶500; Sigma Aldrich) to visualize the ureteric bud and Wolffian duct. Stained urogenital ridges were mounted and imaged using a confocal microscope. CND length from the caudal end of the nephric duct at the cloaca to the cranial edge of the ureteral bud stalk origin was measured using Imaris software (Bitplane, Zurich, Switzerland).

To investigate proliferation in the CND, E12.5 *Fgfr2^ST−/−^* and littermate control embryos were fixed in 4%PFA/PBS, embedded in paraffin and serially sectioned at 10 µm through the urogenital sinus and CND. Sections were then labeled with antibodies against phospho-histone H3 (pH3: 1∶200; Sigma Aldrich) and E-Cadherin (Ecad: 1∶250; Sigma Aldrich) to visualize proliferating cells and the CND respectively. We detected Phospho-histone H3 with goat anti-rabbit Alex Fluor 594 and E-Cadherin with donkey anti-rat Alex Fluor 488 (1∶500; Molecular Probes). A minimum of three sections through the CND, at 20 µm intervals were assessed for each embryo of each genotype.

To examine apoptosis of the CND, methanol fixed E12.5 *Fgfr2^ST−/−^* and littermate control urogential blocks (including mesonephros, metanephros, ureter, gonads and cloaca) were examined using whole mount immunofluorescence with antibodies against pan-cytokeratin (1∶10; Sigma Aldrich) to visualize CNDs and activated caspase-3 (1∶250, Invitrogen) to detect apoptosis, as described previously [22]. We detected cytokeratin with goat anti-mouse Alex Fluor 488 and activated caspase 3 with donkey anti-rabbit Alex Fluor 594 (1∶500; Molecular Probes).

### Cystograms

To assess for VUR, euthanized cystograms were performed in P1 *Fgfr2^ST−/−^* and littermate control pups as described [22]. Briefly, mice were euthanized and abdominal walls were reflected to expose the bladder. A 30-gauge needle was inserted into the bladder and 1% methylene blue dye was gravity filled into the bladder via a 50 ml syringe. Starting at a height of 0 cm, the syringe was raised 30 cm at 5-second intervals to a final height of 150 cm, at which time the syringe was maintained for 15 seconds. The presence of dye ascending from the bladder towards the kidney was determined as VUR. All VUR was graded according to international classifications [23]. At the completion of the assay, all kidneys were photographed for assessment of kidney long axis via Image J software (National Institute of Health).

### Three-Dimensional (3D) Reconstructions

Whole E15.5 embryos and lower body P1 (post-cystogram) tissues from control and *Fgfr2^ST−/−^* mice were fixed in 4% paraformaldehyde overnight at 4°C, dehydrated and paraffin embedded. Tissues were serially sectioned at 10 µm through the ureters and bladder, and stained with hematoxylin and eosin (H&E). Bladders and ureteral insertion sites were reconstructed into three-dimensional models as previously described [10].

### Histology and Immunohistochemistry

All tissues were fixed in 4% paraformaldehyde overnight at 4°C, dehydrated and paraffin embedded. Tissues were sectioned at either 6 or 10 µm and stained with H&E to evaluate tissue morphology. For immunohistochemistry sections were dewaxed, antigen retrieved using citrate buffer, permeabilized using 5% tween in PBS, blocked with 1% donkey serum in PBS, and incubated overnight with alpha smooth muscle actin (αSMA: 1∶250; Sigma Aldrich) and E-cadherin (Ecad: 1∶250; Sigma Aldrich) at 4°C. Sections were then washed and incubated for 1 hour at room temperature with secondary antibodies (Rabbit anti-Mouse 594: 1∶500; Donkey anti-Rat 488: 1∶500; Molecular Probes) and DAPI (1∶10,000) diluted in 1% donkey serum in PBS. Sections were then washed, mounted and visualized.

### Statistics

One-way ANOVA followed by Bonferroni's post-hoc analysis, student's T-tests and fisher's exact test analyses were conducted where appropriate, using GraphPad Prism™ 5 (GraphPad Software Inc, 1992–2007).

## Results

### *Tbx18cre* and *Fgfr2* expression in the peri-Wolffian duct stroma

**Figure 1:**
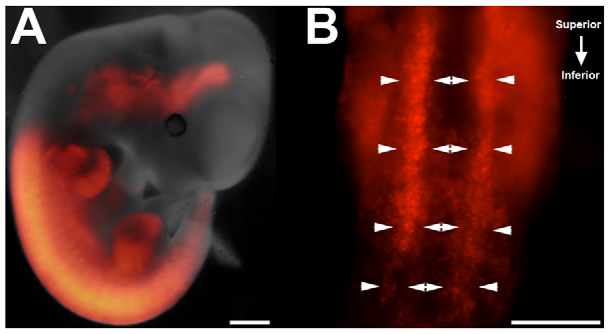
Expression of the Tbx18cre in E10.5 mouse embryos and urogenital tracts. **A.** E10.5 *Cag-Tbx18cre^Tg/+^* mice demonstrate cre expression noted by RFP staining (red) in numerous tissues including fore- and hindlimbs, spine and urogenital ridges. **B.** Ventral view of dissected urogenital ridges illustrates robust cre expression in the peri-Wolffian duct stroma at E10.5 (highlighted between arrowheads). Scale bars = 100 µm. ***p<0.001 vs. control embryos.

**Figure 2:**
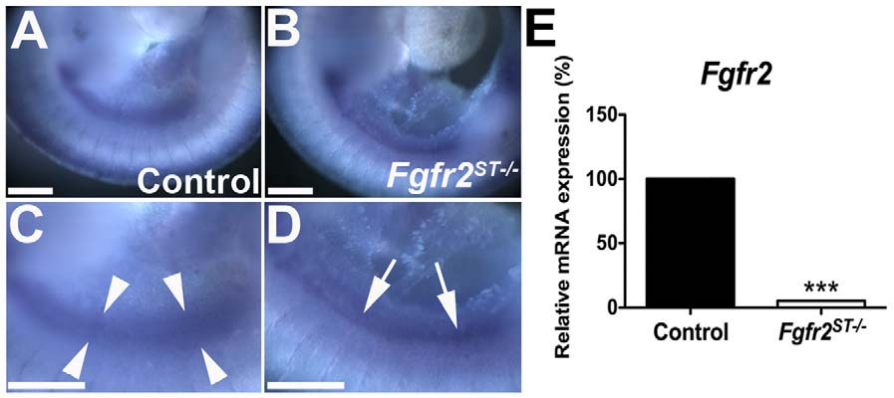
Expression of *Fgfr2* in E10.5 in control and *Fgfr2^ST−/−^* embryos. **A,C.** Lower power (A) and higher power (C) images show that control embryos have a wide band of *Fgfr2* signal (between arrowheads) encompassing the Wolffian duct and surrounding stroma. **B,D**. Lower power (B) and higher power (D) images show that *Fgfr2^ST−/−^* embryos have a linear band of *Fgfr2* expression in the Wolffian duct epithelium (arrows) and not in the surrounding stroma. E. Quantitative real-time PCR of FAC-sorted E10.5 *Cag-Tbx18cre^Tg/+^* (Control) and *Cag-Fgfr2^ST−/−^* (*Fgfr2^St−/−^*) urogenital ridges confirms a dramatic decrease in *Fgfr2* mRNA expression in mutant Tbx18cre expressing cells. Scale bars = 100 µm. ***p<0.001 vs. control embryos.

To confirm expression of the Tbx18cre during ureteric induction deletion we crossed *Tbx18cre* mice with a *CAG:RFP* reporter mouse line (Figure 1A). Robust cre expression was observed in peri-wolffian duct stroma on the dorsal surface of the urogenital tract at E10.5 (Figure 1B). To confirm deletion of *Fgfr2* in peri-Wolffian duct stroma in *Fgfr2^ST−/−^* mice, we performed whole-mount in situ hybridization for *Fgfr2* in E10.5 embryos. In control embryos, a wide band of *Fgfr2* mRNA expression was visualized in both the Wolffian duct and peri-Wolffian duct stroma (Figure 2A, 2C). In *Fgfr2^ST−/−^* embryos, we observed linear *Fgfr2* expression in the Wolffian duct epithelium, but absent expression in the peri-Wolffian duct stroma (Figure 2B, 2D). We also dissected, dissociated, and FACsorted E10.5 *Cag-Tbx18cre^Tg/+^* (control) and *Cag-Fgfr2^ST−/−^* urogenital ridge cells; after isolating mRNA, we confirmed a nearly complete loss of *Fgfr2* expression in the Tbx18cre expressing urogenital ridge cells in *Fgfr2^ST−/−^* mice (5% *Fgfr2* expression in *Fgfr2^ST−/−^* mice relative to controls; Figure 2E). Thus, these data confirm the *Fgfr2^ST−/−^* mice as a model of conditional deletion of *Fgfr2* in the peri-Wolffian duct stroma.

### Aberrant ureteric bud induction following deletion of *Fgfr2* from ST

**Figure 3:**
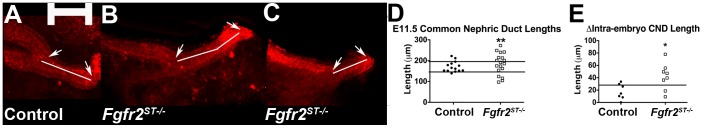
Common nephric duct (CND) lengths in E11.5 control and *Fgfr2^ST−/−^* embryos. **A–C.** Whole mount cytokeratin immunostaining reveals that compared with control common nephric duct lengths (A, line), mutants often have duct lengths that are longer (B, line) or shorter (C, line). Arrows indicate the boundaries of the CND. Scale bar = 100 µm. **D**. Graph demonstrating that the majority of duct lengths in controls are within 1SD of the mean (between the lines), whereas the majority of the *Fgfr2^ST−/−^* lengths are less than or greater than 1SD of the control mean. **E**. Graph demonstrating that the majority of control CND lengths within the same embryo are within one control SD (line), whereas the majority of *Fgfr2^ST−/−^* duct lengths within the same embryo differ by greater than one control SD. *p<0.05, **p<0.01 vs. control embryos.

Previously, deletion of *Fgf2* expression with a transgenic *Pax3cre* line in the MM and peri-Wolffian duct stroma was associated with aberrant UB induction [10]. To determine if deletion of *Fgfr2* in ST is sufficient to induce abnormal ureteric budding, we measured the common nephric duct length in E11.5 urogenital ridges after performing whole mount cytokeratin staining (Figure 3A–C). Mean CND lengths were similar between controls and mutants (Control: 170.75±25.34 µm; *Fgfr2^ST−/−^*: 182.74±53.92 µm; p = 0.5, Figure 3D). However, it appeared that the mutants had a tri-modal distribution of CND lengths as opposed to control CND lengths that appeared to have a Gaussian distribution (Figure 3D). In *Fgfr2^ST−/−^* mice, 69% of the CND lengths were >1 control SD away from the mean (both shorter and longer), whereas only 20% of the control CND lengths were >1 control SD from the mean (p = 0.01). Mutants also had a greater intra-embryo variability in CND length between right and left sides than controls; 75% (6/8) of *Fgfr2^ST−/−^* mice had intra-embryonic CND lengths that were >1 control SD different between the right and left side, whereas only 29% (2/7) of controls had intra-embryonic CND lengths that were >1 SD different between each side (p = 0.04) (Figure 3E). Despite the differences in CND length at E11.5, whole-mount activated caspases-3 staining illustrates similar patterns of ventral apoptosis of the distal CND in both control and *Fgfr2^ST−/−^* urogenital ridges at E12.5 (Figure S1). Similarly, phospho-histone H3 staining of cells in the CND of control and *Fgfr2^ST−/−^* embryos demonstrated a similar percentage of proliferating cells in both genotypes at E12.5 (Figure S2). Together indicating that aberrant ureteric bud induction is not associated with changes in CND apoptosis or proliferation (either decreases or increases) at later stages Thus, deletion of *Fgfr2* from the peri-Wolffian duct stroma induces aberrant UB induction relative to controls.

### *Fgfr2^ST−/−^* embryos have improper ureteral insertion in bladders at E15.5

**Figure 4:**
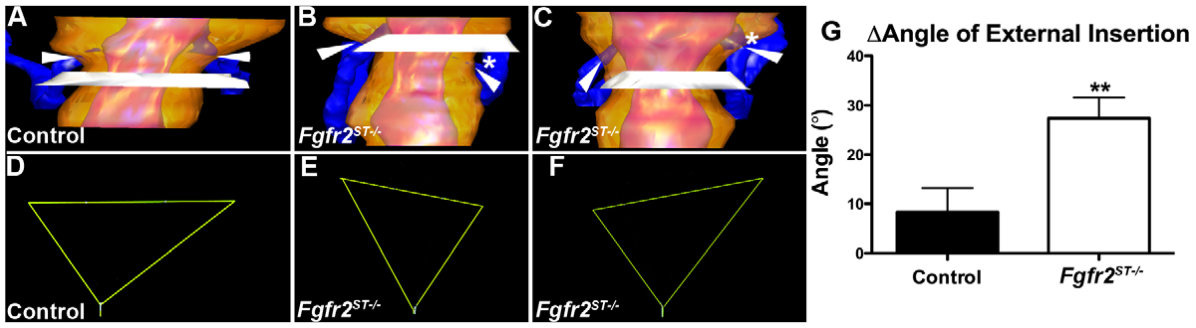
Three-dimensional reconstructions of ureteral insertion sites into the bladders of E15.5 control and *Fgfr2^ST−/−^* embryos. **A** Ureter (blue) insertion sites (arrowheads) in control embryos occur just above the level of the pubic symphysis (white plane) in a symmetric pattern. **B–C**. *Fgfr2^ST−/−^* embryos have asymmetric ureteral insertion into the developing bladder, one with a low left insertion (B, *) and one with a high left insertion (C, *). **D–F**. External trigone of the control (from A) shows a normal inverted isosceles triangle (D), whereas the mutants (from B & C) have distorted external trigones with marked differences in angles at the insertion sites (E, F). **G**. Graph demonstrating the differences in external trigonal angles are much greater in mutants than controls **p<0.01; (n) = 3 per genotype; Values are mean+SD. yellow = bladder surface; pink = bladder lumen.

To determine whether the ureteric induction defects led to abnormal ureteral insertion in the bladder during embryogenesis, we performed H&E staining and 3D reconstructions of E15.5 control and *Fgfr2^ST−/−^* lower urinary tracts. Histologically, the E15.5 ureters and bladders appeared normal (Figure S3). After performing 3D reconstructions (Figure 4), we determined the position of the ureter insertions into the bladder in two ways: First, we generated triangles connecting the two external ureteral insertion sites and the bladder neck (external bladder trigone) from which we could determine whether ureters were displaced. Second, we noted the ureteral insertion site in relation to the plane of the pubic symphysis, which we used as an anatomic reference point.

In control embryos, ureteral insertion occurs just superior to the pubic symphysis, with both ureters inserting into the bladder at a similar anatomical level (Figure 4A). Furthermore, the control external trigones formed nearly perfect inverted isosceles triangles with similar-appearing angles formed at the insertion sites (Figure 4D). In contrast, *Fgfr2^ST−/−^* embryos usually had one displaced ureter including those with low bladder insertions (Figure 4B) and others with high bladder insertions (Figure 4C) relative to the pubic symphysis. The external trigones formed triangles that were distorted from the shape of an isosceles triangle with significant differences between angles formed at the insertion sites (Figure 4E–F). Quantitatively, controls had right and left external angles that were generally within 10° whereas mutants had average differences of approximately 30° (Figure 4G). Thus, E15.5 *Fgfr2^ST−/−^* embryos had evidence of improper unilateral ureteral insertion consistent with the ureteric induction defects noted earlier.

### *Fgfr2^ST−/−^* mice have high incidence of VUR and occasionally other urinary tract anomalies at birth

**Figure 5:**
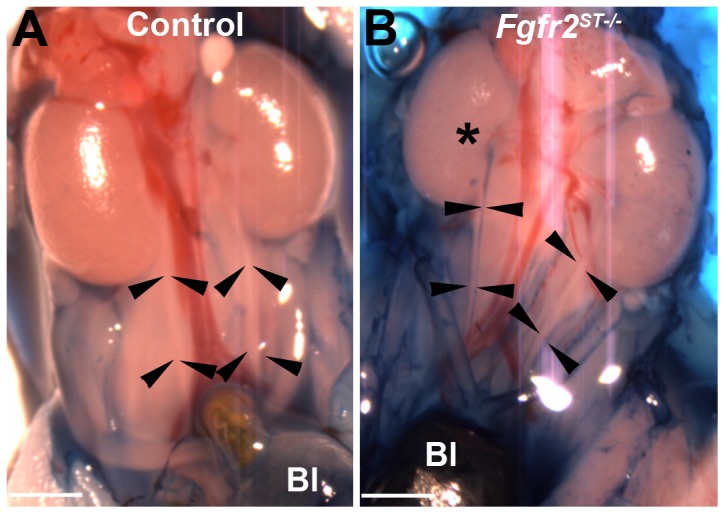
Representative cystograms in P1 *Fgfr2^ST−/−^* and control mice. **A**. Control ureters have no dye (between arrowheads) indicating no VUR. **B**. *Fgfr2^ST−/−^* mouse has dye in the right ureter up to the renal pelvis (*) indicating grade II VUR. Bl indicates the bladder. Scale bar = 500 µm.

Given the evidence of ureteric induction defects and improper ureteral insertion in the bladder in embryos, we assessed P1 *Fgfr2^ST−/−^* mice for VUR with cystograms by gravity filling bladders with methylene blue dye. As shown, P1 *Fgfr2^ST−/−^* mice (excluding those with duplex ureters) had high rates of vesicoureteral reflux relative to control littermates (Figure 5, Table 1). The VUR in *Fgfr2^ST−/−^* mice had no gender bias (Table 1). In addition, mutants usually had unilateral VUR with only one *Fgfr2^ST−/−^* mouse exhibiting bilateral reflux (Table 1). The mutants most frequently had grade II VUR followed by grade III VUR while the few control mice with VUR had grade I reflux (Tables 1 and 2). No VUR greater than grade III was observed in either genotype. Lastly, while *Fgfr2^ST−/−^* mice occasionally had other urinary tract anomalies including duplex collecting systems/kidneys and renal hypoplasia (Figure S4) with an incidence higher than in controls [Control: 0% (0/72) versus *Fgfr2^ST−/−^*: 16% (6/31); p<0.001].

**Table 1:** Incidence and severity of VUR at P1 in control and *Fgfr2^ST−/−^* mice.

Genotype	Incidence of Reflux			Unilateral Reflux Observed		Grade of Reflux	
	Total	Male	Female	Unilateral	Bilateral	Grade I–II	Grade III–V
**Control**	3% 2/76	4% 2/45	0% 0/31	100% 2/2	N/A 0/0	100% 2/2	N/A
***Fgfr2^ST−/−^***	75%*** 23/31	72% 13/18	77% 10/13	96% 22/23	4% 1/23	78% 18/23	22% 5/23

*** p<0.001 vs. control, analysis via a Fisher's Exact Test.

**Table 2:** Grades of VUR at P1 in control and *Fgfr2^ST−/−^* mice.

Genotype	Grade I			Grade II			Grade III		
	Total	Male	Female	Total	Male	Female	Total	Male	Female
**Control**	100% (2/2)	2	0	0	0	0	0	0	0
***Fgfr2^ST−/−^***	9% (2/23)*	1	1	69% (16/23)	9	7	22% (5/23)	3	2

* p<0.05 vs. control, analysis via a Fisher's Exact Test.

### Refluxing P1 *Fgfr2^ST−/−^* mice have improper ureteral insertion and shortened intravesicular tunnel lengths

**Figure 6:**
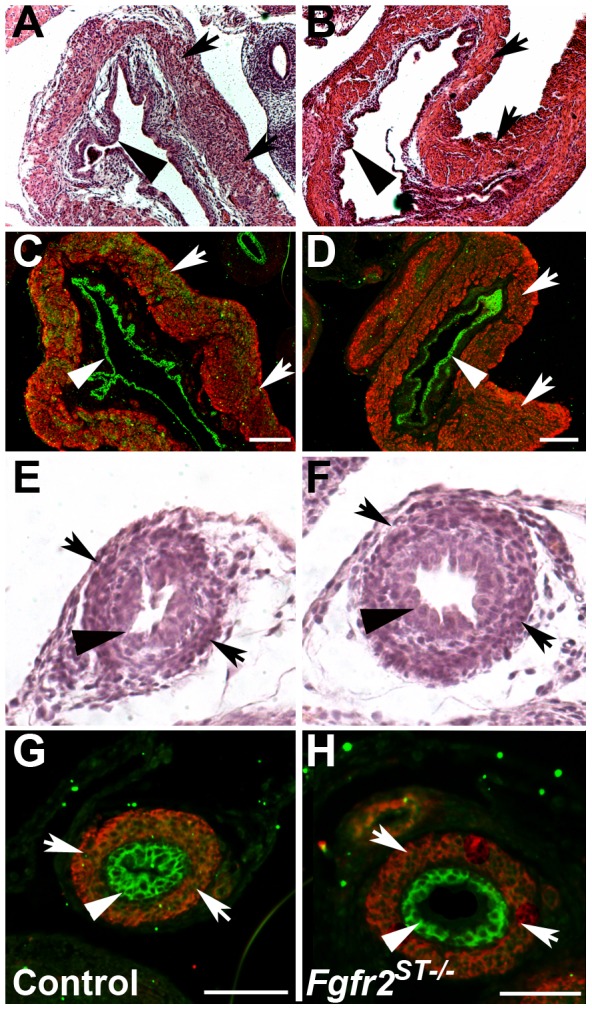
Bladder and ureter morphology in P1 *Fgfr2^ST−/−^* and control mice following cystograms. **A–B.** H&E staining shows similar bladder histology between controls (A) and mutants (B). **C–D**. Immunofluorescence (IF) also shows normal urothelium (green) and alpha smooth muscle actin staining (red) in control (C) and *Fgfr2^ST−/−^* mice (D). **E–H**. H&E staining and IF shows normal urothelium and muscle layers in control ureters (E, G) and mutant ureters (F, H). Arrows indicate muscle layer; Arrowheads indicate urothelium. Scale bar = A–D: 300 µm, E–H: 500 µm.

**Figure 7:**
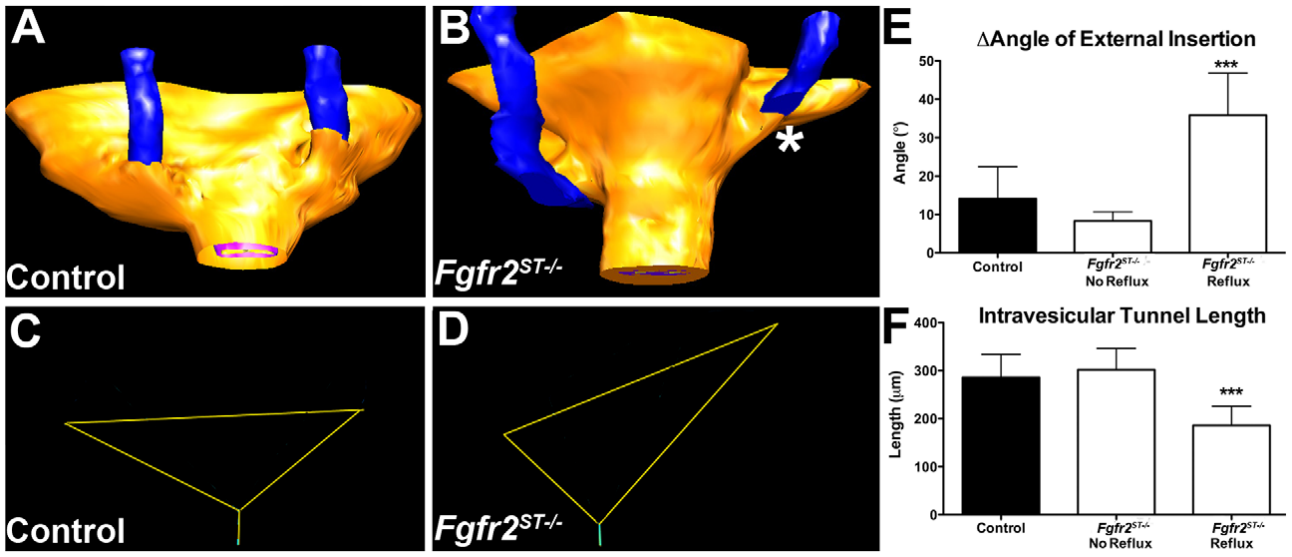
Three-dimensional reconstructions of ureteral insertion sites into the bladders of P1 control and *Fgfr2^ST−/−^* embryos. **A–B**. Control mouse without VUR (A) has ureters (blue) that insert into bladders (yellow) at the same level, whereas the *Fgfr2^St−/−^* mouse with left sided VUR (B) has a high and laterally displaced ureteral insertion site on that side (*). **C–D**. External trigone drawn from the control (from A) shows similar right and left sided external angles whereas the mutant (from B) has a marked differences in the left and right external angles. **E**. Graph demonstrating that the differences in external trigonal angles are much greater in mutants with VUR than controls or mutants without VUR. Values in E–F are mean+SD; ***p<0.001 vs. control and vs. *Fgfr2^St−/−^* mutants without VUR).

We then sought to determine whether there were defects in either urinary tract morphology and/or in ureteral insertion that could explain the high rates of VUR in *Fgfr2^ST−/−^* mice. Thus we performed histological assays and 3D reconstructions of the lower urinary tracts of P1 mice after cystograms. Morphologically, P1 mutant bladders and ureters appeared normal by H&E staining and by immunostaining with markers for muscle and urothelium (Figure 6). However, as in E15.5 mutants, 3D reconstructions revealed abnormal unilateral ureteral insertion sites into the bladders of P1 *Fgfr2^ST−/−^* mice with VUR (including high and low insertions) but normal-appearing insertion sites in mutants without VUR (Figure 7 and not shown). Quantitatively, P1 controls and *Fgfr2^St−/−^* mice without VUR had right and left external angles that were generally within 10° whereas mutants with unilateral reflux had average differences of approximately 35° (Figure 7E). Furthermore, ureters from *Fgfr2^ST−/−^* mice that refluxed had significantly shortened intravesicular tunnel lengths compared with both control and non-refluxing *Fgfr2^ST−/−^* ureters (Control: 286.1±47.92 µm; *Fgfr2^ST−/−^* with no reflux: 302.18±44.41 µm; *Fgfr2^ST−/−^* with reflux: 186.23±39.76 µm; p<0.001) (Figure 7F). Thus the presence of VUR correlated with displaced ureteral insertion sites into the bladder and shorter intravesicular tunnel lengths.

### *Fgfr2* deletion results in decreased *Bmp4* expression in urogenital regions

**Figure 8:**
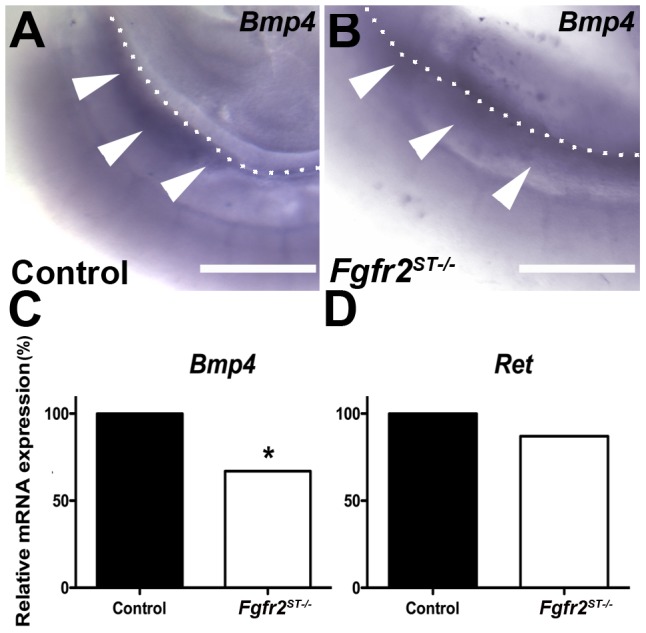
Expression of *Bmp4* and *Ret* mRNA at E10.5 in control and *Fgfr2^ST−/−^* urogenital ridges. **A–B**. In situ hybridization for *Bmp4* shows apparent reduced expression in the peri-Wolffian duct stroma in mutants (B, white arrowheads) versus controls (A, white arrowheads). **C–D**. Graphs of real time PCR confirm that *Fgfr2^ST−/−^* urogenital ridges have an ∼40% reduction in *Bmp4* mRNA (C), whereas *Ret* mRNA levels are equivalent (D). Values are means normalized to control expression levels; *p<0.05, **p<0.01 vs. control; n = 3 urogenital ridges per genotype per analysis. Dotted lines indicate the dorsal aspect of the Wolffian duct in each image. Scale bar = 100 µm.

*Bmp4*, which is secreted by the peri-Wolffian duct stroma, is known to constrain the ureteric bud to its proper induction site along the Wolffian duct [6]. Given that deletion of *Fgfr2* in this tissue leads to defects in ureteric induction, we examined *Fgfr2^ST−/−^* mutants at E10.5 for changes in *Bmp4* and *Ret* expression. Whole mount in situ hybridization at E10.5 illustrated that *Fgfr2^ST−/−^* embryos have significantly less *Bmp4* mRNA expression in the peri-Wolffian duct stroma relative to controls (Figure 8). In contrast, ureteric bud and Wolffian duct expression of *Ret* mRNA appeared similar between both genotypes. Quantitative real-time PCR of dissected urogenital ridges confirmed ∼40% reductions in *Bmp4* mRNA expression in *Fgfr2^ST−/−^* embryos relative to controls while *Ret* expression was equivalent (Figure 8). Thus, the decrease in *Bmp4* expression in *Fgfr2^ST−/−^* embryos correlates with the ureteric induction defects that likely lead to VUR and other phenotypes observed in the mutant mice [6], [7].

## Discussion

Fibroblast growth factor receptors, their ligands and their downstream signaling adapters have previously been shown to be integral for the development of the metanephric kidney, often by conditional deletion in the Wolffian duct/ureteric bud and/or metanephric mesenchyme [9]–[17]. Using a Pax3cre transgenic line, we determined that deletion of *Fgfr2* in both the peri-Wolffian duct stroma and the metanephric mesenchyme led to ureteric induction defects and subsequent VUR and other anomalies such as duplex systems and renal hypoplasia; however, it was unclear as to the contribution of each of these tissues to those defects [9], [10]. By utilizing the *Tbx18cre* mouse line that drives *cre* expression in the peri-Wolffian duct stroma and not in the metanephric mesenchyme, we now conclude that *Fgfr2* expression in the peri-Wolffian duct stroma is most critical for regulating the ureteric bud induction site and preventing VUR and other urinary tract anomalies.

We did observe some differences between *Fgfr2^ST−/−^* mice and *Pax3cre^Tg/+^Fgfr2^Lox/Lox^* mice [10]. First, we detected higher rates of VUR in *Fgfr2^ST−/−^* mice compared to the *Pax3cre* mice; this discrepancy could be due to more complete deletion of *Fgfr2* in the peri-Wolffian duct stroma with the *Tbx18cre* line versus the *Pax3cre* line, as cre expression in the MM has been shown to be variable with the *Pax3cre* line when bred with reporter mice (Drs. Valeria Di Giovanni and Norman Rosenblum, unpublished observations). In addition, we noted shortened intravesicular tunnel lengths and both cranial and caudal displacement of ureteric bud induction sites in *Fgfr2^ST−/−^*, whereas the *Pax3cre* mutant mice had normal tunnel lengths and only cranially displaced ureteric buds. We suspect that we had more power in the current study (larger numbers of mutant mice and higher VUR rates) than the previous study and were therefore able to detect these defects in the *Fgfr2^ST−/−^* mice.

The reason for the VUR in the *Fgfr2^ST−/−^* mice appears to be secondary to ureteric bud induction defects, leading to abnormal ureteric induction sites and incompetent anti-reflux mechanisms, as described by Drs. Mackie and Stephens in humans with VUR [24], [25]. The reflux observed does not appear to be related to a persistence or premature loss of the CND, as apoptosis and proliferation were unaltered in the mutant mice at E12.5. *Fgfr2^ST−/−^* mutants did have evidence of abnormal ureteral insertion sites in the bladder by E15.5; moreover, abnormal position of the ureteral insertion site correlated directly with the presence of VUR in P1 mutants. The P1 mutant ureters with VUR also had shortened intravesicular tunnel lengths, which is known to further increase the risks of VUR in humans [5]. Most of the P1 *Fgfr2^ST−/−^* mice had unilateral VUR, which likely reflects that many of the ureteric bud induction sites at E11.5 were at or near the normal position. We did not detect any histological defects in the ureters or bladders during development or in adult mice to explain the VUR. Finally, we noted other urinary tract anomalies in the mutants including duplex systems and renal hypoplasia, which likely reflect the ureteric induction defects. The high incidence of VUR and the presence of all the aforementioned anatomical features in *Fgfr2^ST−/−^* mice are very consistent with the Mackie-Stephens hypothesis for the developmental origin of VUR in our mice [24], [25].

An important finding of the current study is a likely molecular mechanism responsible for the ureteric induction defects (and associated CAKUTs and VUR) in *Fgfr2^ST−/−^* mice. While we previously found that reduction of *Fgfr* expression in kidney mesenchyme led to increases in *Ret* expression in the ureteric bud [16], we found no evidence of altered *Ret* expression in the UB or Wolffian duct in *Fgfr2^ST−/−^* embryos. Moreover, signaling through *Fgfrs* has been shown to directly and indirectly regulate expression of secreted growth factors, such as Bmp4 [16], [26]. In the current study, *Fgfr2^ST−/−^* mice exhibit a ∼40% reduction in *Bmp4* mRNA expression prior at E10.5, at the onset of ureteric bud induction. Interestingly, in situ hybridization indicated that the entire field of Bmp4 expression seen in control embryos at this stage was significantly reduced in *Fgfr2^ST−/−^* mice. Furthermore, it is possible that this wide area of decreased *Bmp4* expression in the peri-Wolffian duct stroma adjacent to the metanephric mesenchyme may account for the variability in the site of ureteric bud induction observed in *Fgfr2^ST−/−^* mice. Moreover, *Bmp4* heterozygous mice have a 40% reduction in *Bmp4* mRNA levels and have a high incidence of ureteric induction abnormalities [6], [7], [27]. Reduced *Bmp4* mRNA expression as seen in *Bmp4* heterozygous mice or following disruption of upstream regulators of *Bmp4* is strongly associated with urinary tract anomalies such as duplex systems and renal hypoplasia (they have not to our knowledge been tested for VUR) [6], [7], [27], [28]. Findings from our study strongly suggest that *Fgfr2* acts upstream of *Bmp4* in the peri-Wolffian duct stroma to maintain the proper ureteric induction site.

In humans, dysregulation of both FGFRs and BMP4 have been associated with a wide range of urinary tract abnormalities. Non-syndromic kidney and urinary tract anomalies have been associated with mutations in *BMP4* in humans [29], [30]. In addition mutations in FGFRs lead to syndromes including Apert's syndrome, Antley-Bixler syndrome, Pfeiffer syndrome, and Beare-Stevenson syndrome, which are sometimes associated with urogenital anomalies such as hydroureter, solitary kidney, and VUR [31]. Thus it is possible that unidentified mutations in FGFs or FGFRs could affect *BMP4* levels in humans leading to VUR and other urinary tract anomalies; thus, FGF receptors and their ligands are potential candidates for future genetic screening studies in patients with VUR and other urinary tract defects.

## Supporting Information

Figure S1 **Representative images of apoptosis of the CND at E12.5 in control and** ***Fgfr2^ST−/−^*** **embryos.** **A–B.** Co-immunofluorescent labeling of pancytokeratin (red) and activated caspase-3 (green) highlighted a similar pattern of apoptosis (arrowhead) of the distal CND in both control and *Fgfr2^ST−/−^* embryos (the entire CND is between the arrows). Scale bar = 100 µm.
(TIF)

Figure S2 **Cell proliferation in the CND at E12.5 in control and** ***Fgfr2^ST−/−^*** **.** **A–B.** Co-immunofluorescent labeling of phosphor-histone H3 (red; proliferating cells) and E-Cadherin (green; urothelium) illustrates cell proliferation in the CND (arrowhead) in both control and *Fgfr2^ST−/−^* embryos. **C.** Quantification of proliferation in the CND indicates no differences between control and *Fgfr2^ST−/−^* embryos at E12.5. Dotted line indicates CND; Cl – Cloaca. Scale bar = 50 µm.
(TIF)

Figure S3 **E15.5 bladder and ureter morphology in control and** ***Fgfr2^ST−/−^*** **embryos.** **A–B**. H&E stains show similar bladder morphology between control (A) and *Fgfr2^ST−/−^* embryos (B). **C–D.** H&E stains also shows similar ureter morphology between controls (C) and mutants (D). Arrowheads indicate mesenchymal (developing muscular) layer; Arrows indicate urothelium. Scale bars = 300 µm.
(TIF)

Figure S4 **P1** ***Fgfr2^ST−/−^*** **mice with duplex collecting systems/kidneys and renal hypoplasia.** **A.** Control kidney shows normal gross morphology. **B**. *Fgfr2^ST−/−^* mouse has a duplex collecting system filled with dye (arrows) and a duplex kidney (* = two kidney moieties). **C**. *Fgfr2^ST−/−^* mouse has a hypoplastic kidney. Scale bar = 500 µm.
(TIF)
