# Correction: Deletion of Fibroblast Growth Factor Receptor 2 from the Peri-Wolffian Duct Stroma Leads to Ureteric Induction Abnormalities and Vesicoureteral Reflux

**DOI:** 10.1371/journal.pone.0167191

**Published:** 2016-11-18

**Authors:** Kenneth A. Walker, Sunder Sims-Lucas, Valeria E. Di Giovanni, Caitlin Schaefer, Whitney M. Sunseri, Tatiana Novitskaya, Mark P. de Caestecker, Feng Chen, Carlton M. Bates

The Office of Research Integrity at the U.S. Department of Health and Human Services identified concerns regarding the authenticity of data in Fig 2E of the *PLOS ONE* article, "Deletion of Fibroblast Growth Factor Receptor 2 from the Peri-Wolffian Duct Stroma Leads to Ureteric Induction Abnormalities and Vesicoureteral Reflux". The *PLOS ONE* staff discussed this with the authors, who apologized for this matter and replicated the experiment. The replication experiment received oversight from the University of Pittsburgh Office of Research Integrity, who vouches for the veracity of the new figure. A member of *PLOS ONE*'s Editorial Board reviewed and approved the new data, and confirmed that the results and conclusions of the original article are still supported.

In the results section the sixth sentence in the first paragraph should now read: We also dissected, dissociated, and FACsorted E10.5 *Cag-Tbx18cre*^*Tg/+*^ (control) and *Cag-Fgfr2*^*ST-/-*^ urogenital ridge cells; after isolating mRNA, we confirmed a significant loss of *Fgfr2* expression in the Tbx18cre expressing urogenital ridge cells in *Fgfr2*^*ST-/-*^ mice (30% *Fgfr2* expression in *Fgfr2*^*ST-/-*^ mice relative to controls; Fig 2E).

The authors have provided the corrected Fig 2 here.

**Fig 2:**
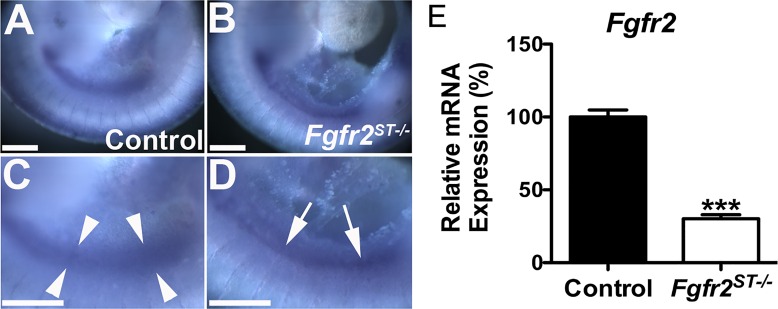
Expression of *Fgfr2* in E10.5 in control and *Fgfr2*^*ST−/−*^ embryos. **A,C.** Lower power (A) and higher power (C) images show that control embryos have a wide band of *Fgfr2* signal (between arrowheads) encompassing the Wolffian duct and surrounding stroma. **B,D.** Lower power (B) and higher power (D) images show that *Fgfr2*^*ST−/−*^ embryos have a linear band of *Fgfr2* expression in the Wolffian duct epithelium (arrows) and not in the surrounding stroma. E. Quantitative real-time PCR of FAC-sorted E10.5 *Cag-Tbx18cre*^*Tg/+*^ (Control) and *Cag-Fgfr2*^*ST−/−*^(*Fgfr2*^*St−/−*^) urogenital ridges confirms a dramatic decrease in *Fgfr2* mRNA expression in mutant Tbx18cre expressing cells. Scale bars = 100 μm. ***p<0.001 vs. control embryos.
