# Supplementary figures and images for: Deletion of Fibroblast Growth Factor Receptor 2 from the Peri-Wolffian Duct Stroma Leads to Ureteric Induction Abnormalities and Vesicoureteral Reflux
Source: PLoS One. 2013 Feb 7;8(2):e56062. doi: 10.1371/journal.pone.0056062 (PMC3567073; doi:10.1371/journal.pone.0056062)

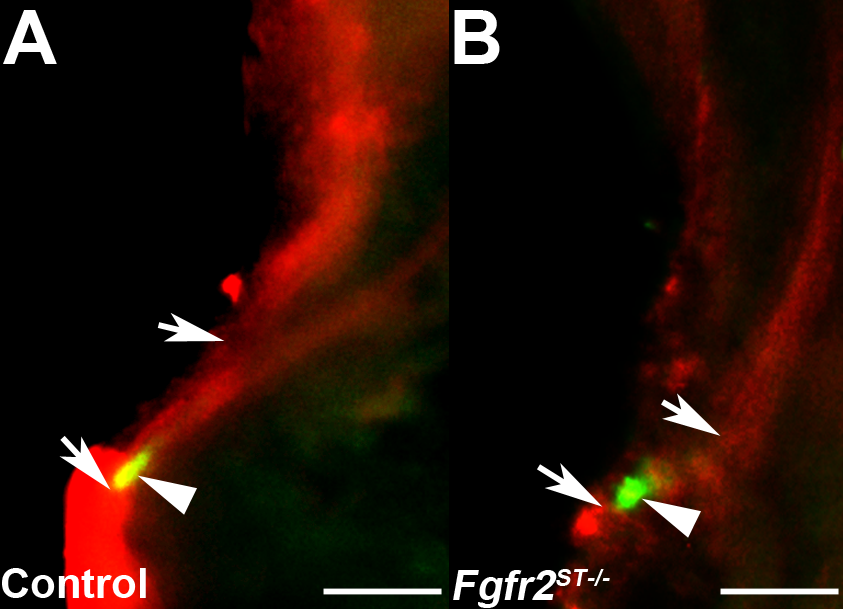

Supplement: Figure S1 — Representative images of apoptosis of the CND at E12.5 in control and Fgfr2ST−/− embryos. A–B. Co-immunofluorescent labeling of pancytokeratin (red) and activated caspase-3 (green) highlighted a similar pattern of apoptosis (arrowhead) of the distal CND in both control and Fgfr2ST−/− embryos (the entire CND is between the arrows). Scale bar = 100 µm. (TIF) [file pone.0056062.s001.tif]

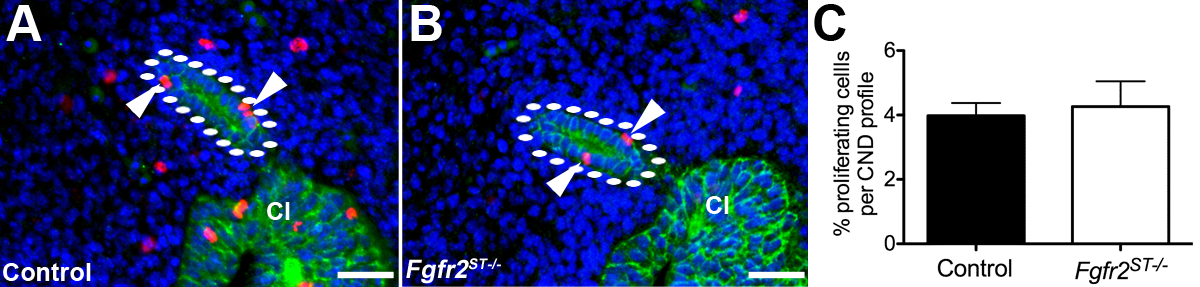

Supplement: Figure S2 — Cell proliferation in the CND at E12.5 in control and Fgfr2ST−/− . A–B. Co-immunofluorescent labeling of phosphor-histone H3 (red; proliferating cells) and E-Cadherin (green; urothelium) illustrates cell proliferation in the CND (arrowhead) in both control and Fgfr2ST−/− embryos. C. Quantification of proliferation in the CND indicates no differences between control and Fgfr2ST−/− embryos at E12.5. Dotted line indicates CND; Cl – Cloaca. Scale bar = 50 µm. (TIF) [file pone.0056062.s002.tif]

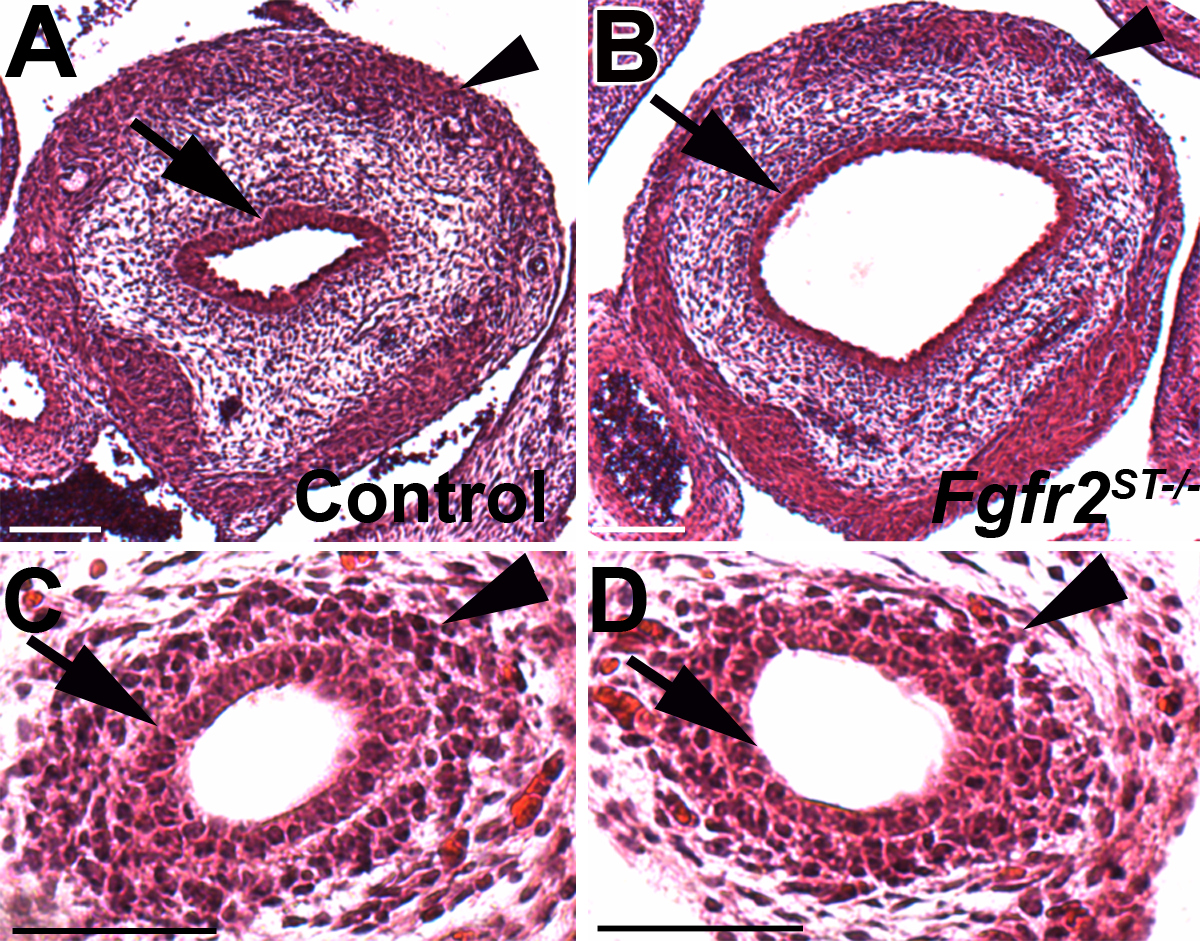

Supplement: Figure S3 — E15.5 bladder and ureter morphology in control and Fgfr2ST−/− embryos. A–B. H&E stains show similar bladder morphology between control (A) and Fgfr2ST−/− embryos (B). C–D. H&E stains also shows similar ureter morphology between controls (C) and mutants (D). Arrowheads indicate mesenchymal (developing muscular) layer; Arrows indicate urothelium. Scale bars = 300 µm. (TIF) [file pone.0056062.s003.tif]

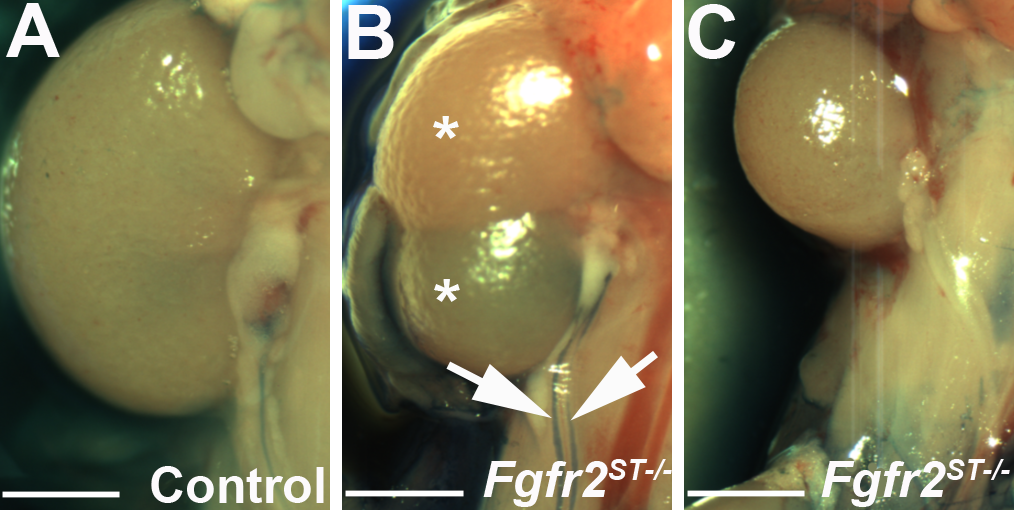

Supplement: Figure S4 — P1 Fgfr2ST−/− mice with duplex collecting systems/kidneys and renal hypoplasia. A. Control kidney shows normal gross morphology. B. Fgfr2ST−/− mouse has a duplex collecting system filled with dye (arrows) and a duplex kidney (* = two kidney moieties). C. Fgfr2ST−/− mouse has a hypoplastic kidney. Scale bar = 500 µm. (TIF) [file pone.0056062.s004.tif]
